# Supplementary material for: Taxonomy of the Genus Bryobia Koch (Acari: Tetranychidae): Reconsideration of Subgenera and Updated Species Groups
Source: Insects. 2024 Nov 3;15(11):859. doi: 10.3390/insects15110859 (PMC11595223; doi:10.3390/insects15110859)
Supplement: Supplementary file 1 [file insects-15-00859-s001.zip › Table S1.pdf]

Table S1. The differential morphological characters of the subgenera in the genus *Bryobia* after Livshits and Mitrofanov (1971) and Mitrofanov (1973)

Subgenus	Morphological Characters						
	**Dorsal Propodosomal lobes**	**Position of setae c3**	**Position of setae f1**	**distance f1-f1 vs f2-f2**	**distance of f1 vs f2**	**duplex on tarsus IV**	**propodosomal lateral angulations**
***Allobia* Livshits and Mitrofanov**	Developed or not, if developed then not deeply incised	Transversely inline with c2	dorsolateral	f1-f1>f2-f2	away	present or absent	absent
***Bryobia* s. str. Livshits and Mitrofanov**	Well developed and separated by deep incision	Transversely inline with c2	dorsolateral	f1-f1>f2-f2	away	present	present
***Bryobiopsis* Mitrofanov**	Absent	Transversely inline with c2	dorsocentral	f1-f1<f2-f2	away	absent	absent
***Eharobia* Livshits and Mitrofanov**	Developed but not deeply incised	Vertically inline with c2	dorsolateral	f1-f1>f2-f2	away	absent	present
***Lyobia* Livshits and Mitrofanov**	Well developed and separated by deep incision	Transversely inline with c2	dorsolateral	f1-f1>f2-f2	away	absent	absent
***Periplonobia* Livshits and Mitrofanov**	Developed or not, if developed then not deeply incised	Transversely inline with c2	dorsosublateral	f1-f1<f2-f2	close	present or absent	absent
