# Supplementary material for: Taxonomy of the Genus Bryobia Koch (Acari: Tetranychidae): Reconsideration of Subgenera and Updated Species Groups
Source: Insects. 2024 Nov 3;15(11):859. doi: 10.3390/insects15110859 (PMC11595223; doi:10.3390/insects15110859)
Supplement: Supplementary file 1 [file insects-15-00859-s001.zip › Table S2.pdf]

Table S2. Exemplary *Bryobia* species with uncertain subgeneric assignment considering Livshits and Mitrofanov (1971) and Mitrofanov (1973) classification

**Species**	**Position of c_3**	**Distance of f_1-f_1 vs f_2-f_2**	**Distance of f_1 from f_2**	**Position of f_1**	**Propod. angulation**	**Duplex on tarsus IV**	**State of lobes**	**Subgeneric Assignment**	**Notes**
Bryobia abyssiniae Fashing and Ueckermann	lateral	f_1-f_1 > f_2-f_2	away	sublateral	absent	absent	from absent to developed	*Allobia* or *Periplonobia*	The position of setae f_1 is sublateral but distance f_1-f_1 is more than f_2-f_2.
Bryobia agioriticus Hatzinikolis and Emmanouel	-	f_1-f_1 > f_2-f_2	away	lateral	absent	present	developed	uncertain	The setae c_3 neither drawn nor illustrated and is assumed to be horizontally aligned with c_2. The species could be designated to either subgenus *Bryobia* or *Lyobia* but could not be designated to any due to absence of lateral angulation and presence of duplex on tarsus IV.
Bryobia apsheronica Khalilova	NOT ILLUSTRATED OR DESCRIBED IN DETAIL						not developed, like tubercles	uncertain	Due to incomplete published data, the subgeneric assignment, proposed in the present study, is not possible.
Bryobia astragali Strunkova & Mitrofanov	lateral	f_1-f_1 > f_2-f_2	away	lateral	absent	present	absent	*Allobia* or *Bryobiopsis*	Original authors placed this species in the subgenus *Allobia*. But it does not fit there due to position of f_1 setae not as in *Bryobiopsis* and lobes absent not as in *Allobia*
Bryobia batrae Baker and Tuttle	sublateral	f_1-f_1 > f_2-f_2	away	lateral	present	present	developed	*Bryobia* or *Eharobia*	This species due to presence of angulations could be designated to subgen. *Bryobia* and due to sublateral c_3 could be designated to *Eharobia*.
Bryobia belliloci Auger, Arabuli & Migeon	lateral	f_1-f_1 > f_2-f_2	close	lateral	absent	absent	developed, not incised	*Periplonobia* or uncertain	Its subgeneric assignment is uncertain because f_1 is contiguous to f_2 but distance is longer.
Bryobia burkei Meyer	lateral	f_1-f_1 > f_2-f_2 (equal to)	away	sublateral	absent	present	developed, not incised	*Allobia* or *Periplonobia*	The character of f_1-f_1 vs f_2-f_2 and position of f_1, places this species in between subgen. *Allobia* and *Periplonobia*.
Bryobia cagani Çobanoğlu, Ueckermann and Cilbircioğlu	lateral	f_1-f_1 > f_2-f_2	away	lateral	absent	present	developed, not incised	*Bryobia* or *Allobia*	Original authors designated it as subgen, *Bryobia* but stated concern on angulation. It could also be designated to *Allobia* subgenus based on shape of lobes
Bryobia calida Karg	NOT ILLUSTRATED NOR DESCRIBED IN DETAIL					present	developed	Uncertain	Lobes are well developed and deeply incised. However, description and illustration is poor hence can not be designated to any subgenus
Bryobia cerasi Hatzinikolis and Emmanouel	lateral	f_1-f_1 > f_2-f_2	away	lateral	present	absent	developed	Uncertain	With lobes well developed and well incised and with lateral angulations, it is suitable for the subgenus *Bryobia* but due to absence of tarsus IV duplex, its status remained uncertain.
Bryobia chongqingensis Ma and Yuan	sublateral	f_1-f_1 > f_2-f_2	away	lateral	absent	absent	developed, not incised	*Eharobia* or uncertain	It is close to *B. eharai* (subgenus *Eharobia*). It could be designated to that subgenus due to lobes developed and not incized and c_3 sublateral, but the angulations are absent, setae f_1 is laterally present.

Bryobia chrysocomae Meyer	lateral	f_1-$f_1 > f_2$-f_2	away, somewhat close	sublateral	absent	absent	developed	uncertain	Due to sublateral position of setae f_1, and well-developed lobes, it is difficult to assign to any existing subgenera.
Bryobia cinereae Auger and Migeon	lateral	f_1-$f_1 > f_2$-f_2	close	sublateral	absent	absent	developed, not incised	uncertain	The subgeneric designation could not be achieved that f_1 and f_2 are contiguous still f_1-f_1>f_2-f_2. Other character and their combinations also could not help to sugbeneric assignment.
Bryobia coatesi Meyer	lateral	f_1-$f_1 > f_2$-f_2	away	lateral	absent	absent	developed, not incised	*Allobia* or *Lyobia*	Due to character of lobes being deeply incised or not, this species could be allocated to *Allobia* or *Lyobia* subgenera.
Bryobia deserticola Meyer	lateral	f_1-$f_1 < f_2$-f_2	away	sublateral	absent	absent	developed, not incised	*Periplonobia* or uncertain	It could be assigned to the subgenus *Periplonobia*, but the setae f_1 and f_2 are not contiguous hence the status is not clear. Also lateral setae appear sublateral.
Bryobia desertorum Hassan, Afifi and Nawar	lateral	f_1-$f_1 > f_2$-f_2	away	lateral	-	-	developed	uncerain	The character of tarsus IV duplex is unclear as is the incision of propodosomal lobes. Hence, it is unclear to place this species either with *Lyobia* or *Allobia*
Bryobia dianthi Mitrofanov and Sharonov	lateral	f_1-$f_1 > f_2$-f_2	away	lateral	absent	absent	developed, unclear	*Lyobia* or uncertain	Designated to subgen. *Lyobia*, but lobes not deeply incized as illustrated, no described as such
Bryobia eharai Pritchard and Keifer	lateral to sublateral	f_1-$f_1 > f_2$-f_2	away	lateral	present	absent	developed	*Bryobia* or *Eharobia*	Placed in subgen. *Eharobia* but lobes and angulation descibed as well developed, *c3* position is not clear, in different published works
Bryobia emmanoueli Hatziniko lis and Panou	lateral	f_1-$f_1 > f_2$-f_2	away	lateral	absent	present	developed	*Bryobia* or uncertain	This species with developed deeply incised lobes and presence of duplex on tarsus IV could be placed in the *Bryobia* subgenus but the anterior angulations are described as absent.
Bryobia fuegina Gonzalez	lateral	f_1-$f_1 > f_2$-f_2	away	lateral	-	present	developed	*Bryobia* or *Lyobia*	Designated under subgen. *Lyobia*, but tarsus IV has duplex present and propodosomal projections are deeply incised as illustrated. Additionally anterior angulations are absent hence could not be assigned to subgenus *Bryobia*
Bryobia gigas Auger, Arabuli and Migeon	sublateral	f_1-$f_1 > f_2$-f_2	away	lateral	absent	present	developed	*Bryobia* or uncertain	Position of c_3, angulation absence, places this species in between the subgenera *Bryobia* and *Eharobia*
Bryobia lucens Meyer	lateral	f_1-$f_1 > f_2$-f_2	away	lateral	present	absent	developed	*Bryobia* or *Lyobia*	It could be placed in the subgenus *Bryobia* due to presence of angulations but also belong to subgenus *Lyobia* due to absence of duplex on tarsus IV.
Bryobia neoribis Tuttle and Baker	lateral	f_1-$f_1 > f_2$-f_2	away	lateral	absent	present	developed	*Bryobia* or *Lyobia*	The original description states the presence of duplex on tarsus IV while later publication states its absence. Also, the propod. projections are described as deeply incised. However, they are not illustrated like that, and anterior angulations are neither described nor illustrated. Hence, this species hangs between *Bryobia*, *Lyobia* and *Allobia* subgenera.
Bryobia orycustodia Meyer	lateral	f_1-$f_1 > f_2$-f_2	away	lateral	absent	absent	developed, not incised	*Allobia* or uncertain	The position of setae f_1 is sublateral but distance f_1-f_1 is more than f_2-f_2. Such a character along with other

									combinations makes this species unable to be assigned to any subgenus.
Bryobia pamirica Mitrofanov	somewhat sublateral	f_1-f_1 < f_2-f_2	close	subcentral to central	absent	absent	developed, not incised	*Periplonobia* or uncertain	It was designated to subgenus *Periplonobia* by Livschitz and Mitrofanov, probably due to sublateral position of setae f_1, however, the setae c_3 are also sublaterally drawn (not described) which is the character of *Eharobia* subgenus.
Bryobia pelerentsi Eyndhoven and Vacante	sublateral	f_1-f_1 > f_2-f_2	away	lateral	absent	absent	developed	*Eharobia* or uncertain	This species is in between *Lyobia* (due to developed incised lobes) and *Eharobia* (due to sublateral position of setae c_3).
Bryobia perinsignis Eyndhoven and Vacante	lateral	f_1-f_1 > f_2-f_2 (equal to)	away	sublateral	absent	absent	developed, not incised	*Periplonobia* or uncertain	Due to its position of f_1 setae, this species could not be placed in any subgenus. The setae f_1 are sublateral to subcentral, far away from f_2 not contiguous, and the distance f_1-f_1>f_2-f=
Bryobia petrilunara Meyer	lateral	f_1-f_1 > f_2-f_2 (equal to)	away	sublateral	absent	present	absent	*Bryobiopsis* or uncertain	Due to shape of lobes/projections, this species fits with *Bryobiopsis* subgenus, but setae f_1 are not central in postion and distance f_1-f_1 is equal or greater than f_2-f_2. Also, tarsus IV has a duplex, which is not the character of the subgenus *Bryobiopsis*.
Bryobia pritchardi Rimando	sublateral	f_1-f_1 > f_2-f_2	away	lateral	-	absent	developed	uncertain	The setae c_3 are present sublaterally, vertically aligned with c_2 which makes it suitable for *Eharobia* subgenus, but other characters do not fit
Bryobia qilianensis Ma and Yuan	lateral	f_1-f_1 > f_2-f_2	away	lateral	absent	present	developed	*Lyobia* or uncertain	The lobes in this species are well developed and illustrated as well incised, the lateral projections are described as absent which makes it suitable for subgenus *Lyobia* but the tarsus IV have duplex setae which are absent in that subgenus. Also setae c_3 are illustrated as vertically aligned with c_2 which is the character of subgenus *Eharobia*.
Bryobia querci Hatzinikolis and Panou	lateral	f_1-f_1 < f_2-f_2	away	central	absent	absent	developed	*Lyobia* or uncertain	The anterior lobes are are well developed and deeply incised without lateral angulations, makes it suitable for *Lyobia* subgenus. However, the setae f_1 are central in position and tarsus IV without duplex making it close subgenus *Bryobiopsis*.
Bryobia strombolii Vacante	lateral	f_1-f_1 > f_2-f_2	away	lateral	present	absent	developed, less incised	*Bryobia* or uncertain	Due to presence of anterior angulations, it could be placed in the subgenus *Bryobia*, but tarsus IV is without duplex, a character state of subgenus *Lyobia*.
Bryobia ribis Thomas	POOR ILLUSTRATIONS AND DESCRIPTIONS							uncertain	The lobes are illustrated as developed and well incised. The other morphological characters are not enough to confidently assign it to a certain subgenus or even genus.
Bryobia spinescens Meyer	lateral	NOT ILLUSTRATED NOR DESCRIBED IN DETAIL				absent	developed, three	uncertain	The well developed lobes, absence of tarsus IV duplex places it in the subgenus *Lyobia*, but the setae f_1 are illustrated sublaterally, a character of subgenus *Periplonobia*.

Bryobia strombolii Vacante	lateral	f_1-f_1 > f_2-f_2	away	lateral	present	absent	developed, less incised	*Bryobia* or uncertain	The lobes are described as low but have been illustrated as deeply incised, lateral angulations are illustrated but not described, the tarsus IV is described and illustrated with duplex, all these characters are not helpful to assign any subgenus
Bryobia triloba Meyer	lateral	f_1-f_1 > f_2-f_2	away	lateral	absent	absent	developed	*Lyobia* or uncertain	Lobes described not deeply incised but illustrated contrary. It could be assigned to *Lyobia* or *Allobia* subgenera.
Bryobia vandaelei Vacante	lateral	f_1-f_1 > f_2-f_2	away	lateral	present	absent	developed	*Bryobia* or uncertain	Not described, but originally illustrated with lateral angulations present, suitable for *Bryobia* subgenus, but tarsus IV is without duplex, a character of subgenus *Lyobia*.
Bryobia vaneyndhoveni Vacante	lateral	f_1-f_1 > f_2-f_2	away	lateral	absent	absent	developed	could be *Lyobia*	The lobes incision is not clear making it in between of *Lyobia* or *Allobia* subgenera.
Bryobia weyerensis Packard	POOR ILLUSTRATIONS AND DESCRIPTIONS							uncertain	Dr. CHW Flecthmann has raised concerns over its family identification.
Bryobia ylikiensis (Hatzinikolis and Emmanouel)	lateral	f_1-f_1 > f_2-f_2	away	lateral	absent	absent	uncertain	could be *Allobia*	The characteristic feature of lobes is not well described making it uncertain for the subgeneric assignment.
