# Supplementary material for: Taxonomy of the Genus Bryobia Koch (Acari: Tetranychidae): Reconsideration of Subgenera and Updated Species Groups
Source: Insects. 2024 Nov 3;15(11):859. doi: 10.3390/insects15110859 (PMC11595223; doi:10.3390/insects15110859)
Supplement: Supplementary file 1 [file insects-15-00859-s001.zip › Table S3.pdf]

Table S3. Morphological features and their states for the subgeneric hierarchical analysis of the *Bryobia* species

Morphological Character	States
**Number of setae on coxa II**	0 = 1
	1 = 2
**Position of *c3***	0 = lateral, transversely inline with setae *c2*
	1 = sublateral, longitudinally inline with setae *c2* and *sc2*
**distance of *f1-f1* vs *f2-f2***	0 = less than
	1 = more than
**distance of *f1* from *f2***	0 = away
	1 = close
**position of *f1***	0 = central
	1 = lateral
	2 = sublateral
**propodosomal angulation**	0 = absent
	1 = present
**duplex on tarsus III**	0 = absent
	1 = present
**duplex on tarsus IV**	0 = absent
	1 = present
**state of prodorsal lobes**	0 = absent
	1 = weakly developed or small lobes
	2 = developed, not deeply incised but shallowly incised
	3 = developed, deeply incised
**Shape of Peritremes**	0 = simple bulb
	1= anastomose of various degree
**Shape of Stylophore**	0 = simple, rounded
	1 = indented
**Length of leg I compare to body length**	0 = equal
	1 = shorter
	2 = longer
**Palp tarsal length to claw length**	0 = shorter or equal
	1 = longer
**Tenent Hairs on Empodium I**	0 = 1 pair
	1 = 2 or more pairs
