# Supplementary material for: Taxonomy of the Genus Bryobia Koch (Acari: Tetranychidae): Reconsideration of Subgenera and Updated Species Groups
Source: Insects. 2024 Nov 3;15(11):859. doi: 10.3390/insects15110859 (PMC11595223; doi:10.3390/insects15110859)
Supplement: Supplementary file 1 [file insects-15-00859-s001.zip › Table S4.pdf]

Table S4. ***Bryobia*** **species and the character states**

Species	Number of setae on Coxae II	Position of c3	Distance of f1-f1 vs f2-f2	Distance of f1 from f2	Position of f1	Propod. Angulation	Duplex on tarsus III	Duplex on tarsus IV	State of lobes	Peritremes	Shape of stylophore	Length of leg I compared to body length	palp tarsus length to claw	Tenent hairs on Emp 1
Pseudobryobia_curiosa (Summers_1953)	1	0	0	0	2	0	0	0	1	0	1	-	0	0
Pseudobryobia_canescens Baker_&_Tuttle_1972	1	0	0	0	0	0	0	0	0	1	0	0	0	0
Pseudobryobia_bakeri McGregor_1950	1	0	0	0	0	0	0	0	0	0	0	0	0	0
Bryobia_abbatielloi (Smiley_&_Baker_1995)	0	0	0	0	0	0	0	0	0	0	1	-	0	0
Bryobia_abyssiniae Fashing_&_Ueckermann_2016	0	0	1	0	2	0	1	1	0	1	1	1	1	1
Bryobia_aegyptiacus (Zaher_Gomaa_&_El-Enany_1982)	0	0	1	0	1	0	0	0	2	1	1	0	0	0
Bryobia_aetnensis_Vacante_1983	0	0	1	0	1	0	1	0	3	1	0	1	1	1
Bryobia_agioriticus Hatzinikolis_&_Emmanouel_1996	0	1	1	0	1	0	1	1	3	1	0	2	0	0
Bryobia_alveolata Auger_&_Flechtmann_2009	0	0	1	0	2	0	1	0	2	1	0	1	1	1
Bryobia_anacantha_Strunkova_and_Mitrofanov_1983	0	0	0	0	0	0	1	0	0	0	0	1	-	0
Bryobia_angolensis Meyer_1987	0	0	1	0	1	0	0	0	3	0	1	0	-	1
Bryobia_angustisetis Jakobashvili_1958	0	1	1	0	1	0	1	0	3	1	-	2	-	0
Bryobia_annatensis Manson_1967	0	0	0	0	2	0	1	0	1	1	-	1	0	1
Bryobia_artemisiae Bagdasarian_1951	0	0	0	1	2	0	1	1	2	1	1	1	0	1
Bryobia_astragali Strunkova_&_Mitrofanov_1983	0	0	1	0	1	0	1	0	0	0	-	1	-	0
Bryobia_attica Hatzinikolis_&_Emmanouel_1990	0	0	1	0	1	1	1	1	3	1	1	-	-	0
Bryobia_bakeri (Zaher_Gomaa_&_El-Enany_1982)	0	0	1	0	1	0	1	1	3	1	0	-	0	0
Bryobia_batrae Baker_&_Tuttle_1994	0	1	1	0	1	1	1	1	3	1	0	-	-	0
Bryobia_beaufortensis Meyer_1992	0	0	1	0	1	0	0	0	1	0	1	1	0	0
Bryobia_belliloci Auger_Arabuli_&_Migeon_2014	0	0	1	1	1	0	1	0	1	1	0	1	0	1

Bryobia_berlesei_Eyndhoven_1957	0	0	1	0	1	0	1	0	3	1	0	1	0	1
Bryobia_birivularis Meyer_1989	0	0	1	0	2	0	0	0	1	0	1	0	-	1
Bryobia_borealis Oudemans_1930	0	0	1	0	1	1	1	1	3	1	1	0	0	1
Bryobia_bucharica Strunkova_&_Mitrofanov_1983	0	0	1	0	1	0	1	0	1	0	-	1	-	0
Bryobia_burkei Meyer_1987	0	0	2	0	2	0	1	1	2	0	1	1	-	1
Bryobia_cagani Çobanoğlu_Ueckermann_&_Cilbircioğlu_2021	0	0	1	0	1	0	1	1	3	1	1	0	0	0
Bryobia_caricae Hatzinikolis_&_Emmanouel_1991	0	0	1	0	1	0	0	0	1	1	1	2	-	0
Bryobia_cavalloroi Vacante_&_Eyndhoven_1986	0	0	1	0	1	0	1	0	3	1	1	1	1	1
Bryobia_centaureae Livshits_&_Mitrofanov_1972	0	0	1	0	1	0	1	0	3	1	-	1	-	0
Bryobia_cerasi Hatzinikolis_&_Emmanouel_1991	0	0	1	0	1	1	1	0	2	1	0	0	-	0
Bryobia_chongqingensis Ma_&_Yuan_1981	0	1	1	0	1	0	1	0	2	-	0	-	-	0
Bryobia_chrysocomae Meyer_1974	0	0	1	2	2	0	1	0	3	1	1	1	-	0
Bryobia_cinereae Auger_&_Migeon_2014	0	0	1	1	2	0	1	0	1	1	0	1	1	1
Bryobia_coatesi Meyer_1974	0	0	1	0	1	0	0	0	2	0	1	1	-	1
Bryobia_confusa_Livschitz_and_Mitrofanov_1966	0	-	-	-	-	-	1	0	3	1	-	1	-	0
Bryobia_convolvulus Tuttle_&_Baker_1964	0	0	2	0	1	0	1	0	2	0	1	1	0	0
Bryobia_cooremani Eyndhoven_&_Vacante_1985	0	0	1	0	1	0	1	0	3	1	-	1	1	1
Bryobia_cyclamenae Hatzinikolis_&_Panou_1996	0	0	1	0	1	0	1	1	3	1	1	2	0	0
Bryobia_dekocki Eyndhoven_&_Vacante_1985	0	0	1	0	1	0	1	0	3	1	-	1	0	1
Bryobia_deserticola Meyer_1989	0	0	0	0	2	0	0	0	2	1	1	1	-	0
Bryobia_dianthi Mitrofanov_&_Sharonov_1983	0	0	1	0	1	0	1	0	2	1	-	1	1	0
Bryobia_dikmenensis Eyndhoven_&_Vacante_1985	0	0	1	0	1	0	1	0	2	1	-	1	1	0
Bryobia_dubinini Bagdasarian_1960	0	0	1	0	1	0	1	0	2	1	1	2	0	0
Bryobia_eharai Pritchard_&_Keifer_1958	0	1	1	0	1	1	1	0	2	1	1	0	0	0
Bryobia_emmanoueli Hatzinikolis_&_Panou_1996	0	0	1	0	1	0	1	1	3	1	1	1	0	0
Bryobia_ericoides Meyer_1974	0	0	0	0	0	0	1	0	2	1	1	1	-	0

Bryobia_eurotiae Mitrofanov_1973	0	0	0	0	0	0	1	0	0	1	-	0	-	0
Bryobia_exserta Wang_1985	0	0	1	0	1	1	1	1	3	1	1	2	0	0
Bryobia_fuegina_Gonzalez_1977	0	0	1	0	1	0	1	1	3	1	1	1	0	0
Bryobia_geigeriae Meyer_1974	0	0	1	0	1	0	1	1	1	0	1	1	-	1
Bryobia_geyeri Meyer_1974	0	0	1	0	1	0	0	0	2	1	1	0	0	0
Bryobia_giannitsensis Hatzinikolis_&_Panou_ 1996	0	0	1	0	1	0	0	0	2	1	1	1	0	0
Bryobia_gigas Auger_Arabuli_&_Migeon_20 14	0	1	1	0	1	0	1	1	3	1	-	0	0	0
Bryobia_glacialis Berlese_1913	0	1	1	0	1	1	1	1	3	1	0	1	0	1
Bryobia_graminum (Schrank_1781)	0	0	1	0	1	1	1	1	3	1	-	1	-	0
Bryobia_gushariensis Livshits_&_Mitrofanov _1972	0	0	1	0	1	0	1	0	2	1	-	0	-	0
Bryobia_hengduanensis Wang_&_Cui_1991	0	0	1	0	1	0	1	1	2	1	1	1	0	1
Bryobia_imbricata Meyer_1974	0	0	1	0	1	0	0	0	3	1	1	1	-	1
Bryobia_incana Meyer_1992	0	0	1	0	1	0	0	0	2	0	1	1	0	1
Bryobia_japonica Ehara_&_Yamada_1968	0	0	1	0	1	0	1	1	0	1	-	-	0	1
Bryobia_kakamaensis Meyer_1987	0	0	1	0	1	0	0	0	3	0	0	2	-	1
Bryobia_kakuliana Reck_1956	0	0	1	0	1	0	1	0	2	0	1	1	-	1
Bryobia_karooensis Meyer_1974	0	0	1	0	1	0	1	1	1	1	1	1	0	0
Bryobia_kassioticus Hatzinikolis_&_Panou_1 997	0	0	1	0	1	0	1	0	3	1	1	1	0	0
Bryobia_kissophila Eyndhoven_1955	0	0	1	0	1	1	1	1	3	1	0	2	0	0
Bryobia_latisetae Wang_1985	0	0	1	0	1	1	1	1	3	1	1	-	0	0
Bryobia_longisetis Reck_1947	0	0	1	0	1	0	1	0	1	1	0	-	0	1
Bryobia_lonicerae Reck_1956	0	0	1	0	1	0	1	0	3	1	-	1	-	0
Bryobia_lucens Meyer_1974	0	0	1	0	1	1	0	0	3	1	1	0	-	0
Bryobia_macedonica Hatzinikolis_&_Panou_ 1996	0	0	1	0	1	0	0	0	1	1	1	1	0	0
Bryobia_macrotibialis Mathys_1962	0	0	1	0	1	1	1	1	3	1	-	2	-	0
Bryobia_magallanica Gonzalez_1977	0	0	1	0	1	1	1	1	3	1	0	1	0	0
Bryobia_marcandrei Hatzinikolis_&_Panou_1 996	0	0	1	0	1	0	0	0	3	1	1	2	0	0
Bryobia_mercantourensis Auger_&_Migeon_ 2014	0	0	1	0	1	0	1	0	3	1	-	1	1	0

Bryobia_meteoritica Hatzinikolis_&_Panou_1996	0	0	1	0	1	1	1	1	3	1	1	1	0	0
Bryobia_meyerae Zaher_Gomaa_&_El-Enany_1982	0	0	1	0	1	0	0	0	2	0	1	-	0	0
Bryobia_mirmoayedii Khanjani_Gotoh_&_Kitashima_2008	0	0	1	0	1	0	1	0	2	1	0	1	0	1
Bryobia_monechmae Meyer_1974	0	0	1	0	1	0	0	0	2	1	1	1	-	1
Bryobia_montana Mitrofanov_1973	0	0	1	0	1	0	1	1	1	-	-	1	-	0
Bryobia_monticola Wang_1985	0	0	1	0	1	1	1	1	3	1	1	2	0	0
Bryobia_nasrvasensis Bagdasarian_1960	0	0	0	1	2	0	1	0	1	1	1	-	0	1
Bryobia_neoephedrae (Gutierrez_&_Bolland_1998)	0	0	0	0	0	0	1	1	0	1	0	1	-	-
Bryobia_neopraetiosa Meyer_1974	0	0	1	0	1	1	1	1	3	1	0	1	0	0
Bryobia_praetiosa Koch_1836	0	0	1	0	1	1	1	1	3	1	1	2	0	0
Bryobia_pritchardi_Rimando_1962	0	0	1	0	1	0	0	0	3	1	0	2	0	1
Bryobia_rubrioculus (Scheuten_1857)	0	0	1	0	1	0	1	0	3	1	1	0	0	0
Bryobia_sarothamni Geijskes_1939	0	0	0	1	2	0	1	0	1	1	0	1	0	1
Bryobia_pritchardi_Rimando_1962	0	1	1	0	1	0	0	0	3	1	0	2	0	1
