# Supplementary material for: Taxonomy of the Genus Bryobia Koch (Acari: Tetranychidae): Reconsideration of Subgenera and Updated Species Groups
Source: Insects. 2024 Nov 3;15(11):859. doi: 10.3390/insects15110859 (PMC11595223; doi:10.3390/insects15110859)
Supplement: Supplementary file 1 [file insects-15-00859-s001.zip › Table S5.pdf]

Table S5. Some morphological features of the *Bryobia* species described by Meyer (1974, 1987) having padlike/straight true claws

Species	Host Plant	Country	Legs										
			Leg I Length	Emp. Tenent		Coxae				Femur			
				I	II-IV	I	II	III	IV	I	II	III	IV
Bryobia ericoides Meyer, 1974	*Eriocephalus ericoides*	South Africa	shorter than body	one pair	two rows	-	-	-	-	16	9	5	5
Bryobia geigeriae Meyer, 1974	*Geigeria rigida, Senecio glutinosus, Geigeria ornativa, Geigeria aspera*	South Africa	shorter than body	two rows	two rows	-	-	-	-	11	10	5	5
Bryobia karooensis Meyer, 1974	*Aptosimum marlothii, Atriplex semibaccata, Chrysocoma coma-aurea, C. oblongifolia, Diospyros lucioides, Felicia muricata, Galenia procumbens, Melolobium candicans, Pentzia globosa, Phaeoptilum spinosum*	South Africa	shorter than body	four pairs	two rows	2	1	1	1	10/12	8	5	5
Bryobia lucens Meyer, 1974	*Petalidium lucens*	South Africa.	shorter than body	one pair	-	-	-	-	-	16-18	10	6	6
Bryobia rhodesiana Meyer, 1974	*Acanthaceae sp., Amaranthus hybridus, Diospyros lycioides, Ormocarpum kirkii*	Zimbabwe	shorter than body	one pair	four pairs	-	-	-	-	15	8	5	5
Bryobia spinescens Meyer, 1974	*Amphiglossa triflora, Helichrysum pentzioies*	South Africa	shorter than body	one pair	two rows	2	1	1	1	12/13	8	4	5
Bryobia triloba Meyer, 1974	*Barleria monticola, Thunbergia dregeana, Convolvulus farinosus, Ipomoea oblongata, Poaceae sp. and Lippia rehmannii*	South Africa	shorter than body	one pair	two rows	-	-	-	-	18/20	11	5	5
Bryobia kakamaensis Meyer, 1987	*Barleria rigida*	South Africa	longer than body	four pairs	two rows	2	1	1	1	17	11	6	5

Species	Legs												Spermatheca
	Genu				Tibiae				Tarsus				
	I	II	III	IV	I	II	III	IV	I	II	III	IV	
Bryobia ericoides Meyer, 1974	8	5	5	5	13(1)	9	9	9	19(4) + 2 dup	14-15(1) + 1 dup	13+ 1 dup	14(1)	Not found
Bryobia geigeriae Meyer, 1974	8	5	6	6	13(1)	9	9	9	20(3)+2	15(1) + 1 dup	13 + 1 dup	13 + 1 dup	sausage like

Bryobia karooensis Meyer, 1974	8	5	5	5	13(1)	9	9	9	19(3)+2	15(1) + 1 dup	13 + 1 dup	13 + 1 dup	sausage like
Bryobia lucens Meyer, 1974	8	5	5	5	15(1)	9	9	9	19(3)+2	15 + 1 dup	14(1)	14(1)	small oval
Bryobia rhodesiana Meyer, 1974	8	5	5	5	15(1)	9	9	9	19(5)+2dup	15(2) + 1dup	14(1)	14(1)	oval
Bryobia spinescens Meyer, 1974	8	5	5	5	14(1)	9	9	9	19(3)+2dup	15(1) + 1dup	14(1)	14(1)	small oval
Bryobia triloba Meyer, 1974	8	6	6	6	15(1)	9	9	9	19(6)+2dup	15(2) + 1dup	14(1)	14(1)	small rounded
Bryobia kakamaensis Meyer, 1987	8	5	5	5	15(1)	10	9	9	17/19(5)+2dup	15(1) + 1dup	14(1)	14(1)	sausage like
